# MR Imaging in Ataxias: Consensus Recommendations by the Ataxia Global Initiative Working Group on MRI Biomarkers

**DOI:** 10.1007/s12311-023-01572-y

**Published:** 2023-06-06

**Authors:** Gülin Öz, Sirio Cocozza, Pierre-Gilles Henry, Christophe Lenglet, Andreas Deistung, Jennifer Faber, Adam J. Schwarz, Dagmar Timmann, Koene R. A. Van Dijk, Ian H. Harding, AGI Working Group on MRI Biomarkers, Astrid Adarmes-Gomez, Andreas Thieme, Kathrin Reetz, Marcin Rylski, Thiago JR Rezende, Vincenzo A. Gennarino, Eva-Maria Ratai, Caterina Mariotti, Anna Nigri, Lorenzo Nanetti, Martina Minnerop, Sylvia Boesch, Elisabetta Indelicato, Chiara Pinardi, Kirsi M Kinnunen, Niccolo Fuin, Alexander Gussew, Cherie Marvel, James Joers

**Affiliations:** 1 Center for Magnetic Resonance Research, Department of Radiology, University of Minnesota, 2021 Sixth Street Southeast, Minneapolis, MN 55455 USA; 2 UNINA Department of Advanced Biomedical Sciences, University of Naples Federico II , Naples, Italy; 3 Department for Radiation Medicine, University Clinic and Outpatient Clinic for Radiology, University Hospital Halle (Saale), Halle (Saale), Germany; 4 German Center for Neurodegenerative Diseases (DZNE), Bonn, Germany; 5 Department of Neurology, University Hospital Bonn, Bonn, Germany; 6 Takeda Pharmaceuticals Ltd., Cambridge, MA USA; 7 Department of Neurology and Center for Translational Neuro- and Behavioral Sciences (C-TNBS), Essen University Hospital, University of Duisburg-Essen, Essen, Germany; 8 Digital Sciences and Translational Imaging, Early Clinical Development, Pfizer, Inc., Cambridge, MA USA; 9 Department of Neuroscience, Central Clinical School, Monash University, Melbourne, Australia; 10 Monash Biomedical Imaging, Monash University, Melbourne, Australia; 11 Instituto de Biomedicina de Sevilla, Hospital Universitario Virgen del Rocio/CSIC/Universidad de Sevilla, Sevilla, Spain; 12 Centro de Investigacion Biomedica en Red sobre Enfermedades Neurodegenerativas (CIBERNED), Madrid, Spain; 13 Department of Neurology, RWTH Aachen University, Aachen, Germany; 14 JARA Institute Molecular Neuroscience and Neuroimaging, Forschungszentrum Julich GmbH and RWTH Aachen University, Aachen, Germany; 15 Department of Radiology, Institute of Psychiatry and Neurology (IPiN), Warsaw, Poland; 16 Department of Neurology, School of Medical Sciences, University of Campinas (UNICAMP), Campinas, Brazil; 17 Department of Genetics & Development, Columbia University Irving Medical Center, New York, NY USA; 18 Columbia Stem Cell Initiative, Columbia University Irving Medical Center, New York, NY USA; 19 Department of Pediatrics, College of Physicians & Surgeons, Columbia University Irving Medical Center, New York, NY USA; 20 Department of Neurology, Columbia University Irving Medical Center, New York, NY USA; 21 Initiative for Columbia Ataxia and Tremor, Columbia University Irving Medical Center, New York, NY USA; 22 Massachusetts General Hospital, Department of Radiology, Harvard Medical School, A. A. Martinos Center for Biomedical Imaging, Charlestown, MA USA; 23 Fondazione IRCCS Istituto Neurologico Carlo Besta, Milan, Italy; 24 Neuroradiology Unit, Fondazione IRCCS Istituto Neurologico Carlo Besta, Milan, Italy; 25 Institute of Neuroscience and Medicine (INM-1), Research Centre Juelich, Juelich, Germany; 26 Institute of Clinical Neuroscience and Medical Psychology, Medical Faculty & University Hospital Dusseldorf, Heinrich Heine University Dusseldorf, Dusseldorf, Germany; 27 Department of Neurology, Center for Movement Disorders and Neuromodulation, Medical Faculty & University Hospital Dusseldorf, Heinrich Heine University Dusseldorf, Dusseldorf, Germany; 28 Center for Rare Movement Disorders Innsbruck, Department of Neurology, Medical University of Innsbruck, Innsbruck, Austria; 29 Health Physics Unit, ASST Nord Milano, Milan, Italy; 30 IXICO, London, UK; 31 University Clinic and Outpatient Clinic for Radiology, University Hospital Halle (Saale), Halle (Saale), Germany; 32 Department of Neurology, Johns Hopkins University School of Medicine, Baltimore, MD USA

**Keywords:** MRI biomarkers, Structural MRI, Magnetic resonance spectroscopy, Diffusion MRI, Quantitative susceptibility mapping, Functional MRI

## Abstract

With many viable strategies in the therapeutic pipeline, upcoming clinical trials in hereditary and sporadic degenerative ataxias will benefit from non-invasive MRI biomarkers for patient stratification and the evaluation of therapies. The MRI Biomarkers Working Group of the Ataxia Global Initiative therefore devised guidelines to facilitate harmonized MRI data acquisition in clinical research and trials in ataxias. Recommendations are provided for a basic structural MRI protocol that can be used for clinical care and for an advanced multi-modal MRI protocol relevant for research and trial settings. The advanced protocol consists of modalities with demonstrated utility for tracking brain changes in degenerative ataxias and includes structural MRI, magnetic resonance spectroscopy, diffusion MRI, quantitative susceptibility mapping, and resting-state functional MRI. Acceptable ranges of acquisition parameters are provided to accommodate diverse scanner hardware in research and clinical contexts while maintaining a minimum standard of data quality. Important technical considerations in setting up an advanced multi-modal protocol are outlined, including the order of pulse sequences, and example software packages commonly used for data analysis are provided. Outcome measures most relevant for ataxias are highlighted with use cases from recent ataxia literature. Finally, to facilitate access to the recommendations by the ataxia clinical and research community, examples of datasets collected with the recommended parameters are provided and platform-specific protocols are shared via the Open Science Framework.

**Supplementary Information:**

The online version contains supplementary material available at 10.1007/s12311-023-01572-y.

## Introduction

The last decade has witnessed promising new developments in disease-modifying therapies for degenerative ataxias [1, 2]. Numerous potential strategies at gene, transcript, and protein levels, as well as therapies targeting downstream pathways, are in the therapeutic pipeline for hereditary and sporadic ataxias. The success of such trials will be facilitated by validated biomarkers that inform patient selection or stratification and/or the response to therapies (i.e., pharmacodynamic biomarkers) beyond clinical assessments. MRI biomarkers provide objective biological readouts of neurodegeneration, including the early stages before clinical onset [3]. Non-invasive MRI outcomes will therefore aid therapy evaluation and efficient trial design in upcoming multi-institutional clinical trials in these rare diseases [4]. Prospective longitudinal studies will be particularly important to validate MRI biomarkers for clinical trial readiness. While several multi-site longitudinal imaging studies are ongoing in common degenerative ataxias [5, 6], the majority of the MRI studies thus far have demonstrated cross-sectional group differences, and more longitudinal studies are needed to evaluate the sensitivity of MR biomarkers to progressive pathology [3].

To facilitate harmonized data acquisition in clinical research and trials in ataxias, the guidelines described in this manuscript were prepared by a core group of the Ataxia Global Initiative (AGI) [7] Working Group on MRI Biomarkers and endorsed by collaborating members of the working group who are listed as Study Group Authors. The author group includes global representation from 19 academic institutions and 3 companies. In developing the guidelines, we reviewed standardized MRI protocols of other consortia [8–11] and incorporated the input of imaging experts outside the ataxia domain. The key guiding principles in developing the consensus recommendations were (1) inclusivity of diverse scanner hardware and research/clinical contexts while maintaining a minimum standard of data quality and (2) utilizing existing optimized MR data acquisition protocols.

Recommendations are provided for a basic and an advanced protocol, with 3 tesla (T) magnetic field strength as the preferred platform for both protocols (Table 1). The basic protocol contains T1- and T2-weighted structural MRI sequences that (i) are commonly acquired in clinical settings and (ii) are most likely to be broadly relevant to most ataxia research, clinical trials, and pooled multi-site data analyses. The advanced protocol contains additional sequences that (i) are commonly acquired in research settings, (ii) have demonstrated utility for describing and/or tracking brain changes in ataxias based on currently available literature, and (iii) may be more relevant to targeted research questions in selected ataxias. These include MR spectroscopy (MRS), quantitative susceptibility mapping (QSM), diffusion MRI (dMRI), and resting-state functional MRI (rs-fMRI). The detailed rationale for the inclusion of each modality is outlined in the respective sections below. These advanced modalities may be selected in different ataxia studies/trials based on the specific research question or mechanism of action of the tested therapeutic intervention, as well as technical feasibility at participating sites. The exclusion of other imaging modalities (e.g., positron emission tomography, perfusion MRI, contrast-enhanced MRI) from these guidelines should not be taken as a statement by the Working Group on their relative utility in ataxia research settings, but rather an indication that there is not yet an established evidence base using these techniques.

**Table 1:** Overall guidelines for AGI MRI protocol

Field strength	3 T preferred if available, 1.5 T acceptable for basic protocol
RF coil	Body coil transmit, multi-channel receive array (12–64 channel coils)
Coverage	Whole brain, ensuring entire cerebellum coverage
Basic protocol	3D-T1w volume, 1 mm isotropic @1.5 T or 0.8 mm isotropic @3 T 3D-T2w volume, 1 mm isotropic @1.5 T or 0.8 mm isotropic @3 T
Advanced protocol (only at 3 T)	3D-T1w volume 3D-T2w volume MR spectroscopy (MRS) Quantitative susceptibility mapping (QSM) Diffusion MRI (dMRI) Resting-state functional MRI (rs-fMRI)

To maximize utility and inclusivity, the AGI MRI protocol specifies acceptable ranges of parameters, alongside examples of “ideal” protocols for certain scanners (see Open Science Framework collection: https://osf.io/af46y/?view_only=82d605af57ec477b9ca8ba8f2404239c). This approach was chosen after evaluation of the trade-offs between a fully harmonized protocol and a constrained protocol. Full harmonization with fixed parameters would minimize variability in image properties, image quality, and outcome measure values. While many multi-site studies attempt to harmonize the acquisition parameters as much as possible, usually by means of centralized direction and oversight (e.g., a sponsored clinical trial or natural history study), full harmonization is usually impractical unless only a very selected set of identical or highly compatible scanners is used. In the context of AGI, full harmonization would thus limit participation to a subset of sites interested in global ataxia initiatives that can adhere to the proposed protocol, which is undesirable, particularly in the context of rare diseases. Retrospective data harmonization (e.g., ComBat [12]) or statistical correction approaches (e.g., linear mixed modelling) are now regularly employed in multi-site studies to address issues engendered by variability in image acquisitions. For any clinical research study where full harmonization is not possible, we recommend the acquisition of data from an age- and sex-matched normative control group at each participating site. If the collection of such control data is not feasible, site-to-site variability can be accommodated by including the site as a covariate in the statistical model given comparable cohort characteristics across sites.

The following sections outline our recommendations for the selected MR modalities with a primary focus on the brain, with special considerations about the spinal cord included where appropriate. An illustrative selection of software packages commonly used for image analysis in the academic environment is provided for the various sequence types; however, proprietary image analysis software based on these and similar algorithms, implemented within auditable and regulatory agency-compatible (e.g., CFR 21.11) environments, are also available as services from commercial imaging core laboratories for industry-sponsored trials.

When implementing a multi-modal protocol on the MR scanner, we recommend using commercially available tools such as AutoAlign (Siemens) and SmartExam (Philips) to allow the collection of all images in the same reference frame in all subjects/sessions. When collecting multiple advanced sequences (QSM, dMRI, rs-fMRI), we recommend prescribing the same field of view (FoV) for each acquisition, ensuring full coverage of the cerebellum (Supplementary Fig. 1). In addition, we recommend the order of pulse sequences shown in Table 1 considering (1) participant movement increases with scan time; **(**2) T1, T2, and QSM are 3D sequences and are therefore severely affected by subject motion, whereas dMRI and rs-fMRI are fast acquisitions, for which motion can be accounted for to a certain extent by data processing approaches; (3) gradient heating after dMRI results in frequency drift on some scanners, which diminishes localization accuracy and hampers water suppression in MRS; (4) while both QSM and MRS are sensitive to motion during the acquisition, MRS is also sensitive to motion between the anatomical scan (used for prescribing the volume of interest (VOI)) and the start of the MRS scan. If QSM is prioritized before MRS, an additional highly accelerated T1 scan can be acquired before MRS to prescribe the VOI.

## Morphometry

Progressive brain tissue loss is a hallmark of almost all neurodegenerative disorders, and structural MRI represents the best method to obtain an accurate measurement of this phenomenon in vivo, allowing for the quantification of volumes of cortical and subcortical structures and their changes over time.

Volumetric assessments primarily utilize T1-weighted (T1w) gradient-echo images. These sequences provide excellent contrast between relatively bright parenchyma and dark cerebrospinal fluid (CSF), between grey matter and white matter, and are widely accepted as the standard approach to evaluate brain atrophy. T2-weighted (T2w) turbo/fast-spin-echo images are additionally useful for the evaluation of possible pathological signal changes affecting the cerebellum and brainstem [13]. For example, when used in conjunction with T1w images, T2w images improve cortical thickness evaluations, especially at the subpial level [14], and allow for more accurate brain masking through the exclusion of meninges and macrovasculature. Also, T2w images allow assessment of additional white matter disease, and the T1w/T2w ratio may be used as a proxy of intracortical myelination [15]. To facilitate co-registration of T1w and T2w images and full brain coverage, 3D T2w volumes should be acquired with the same spatial resolution and orientation of the T1w counterpart.

**Fig. 1:**
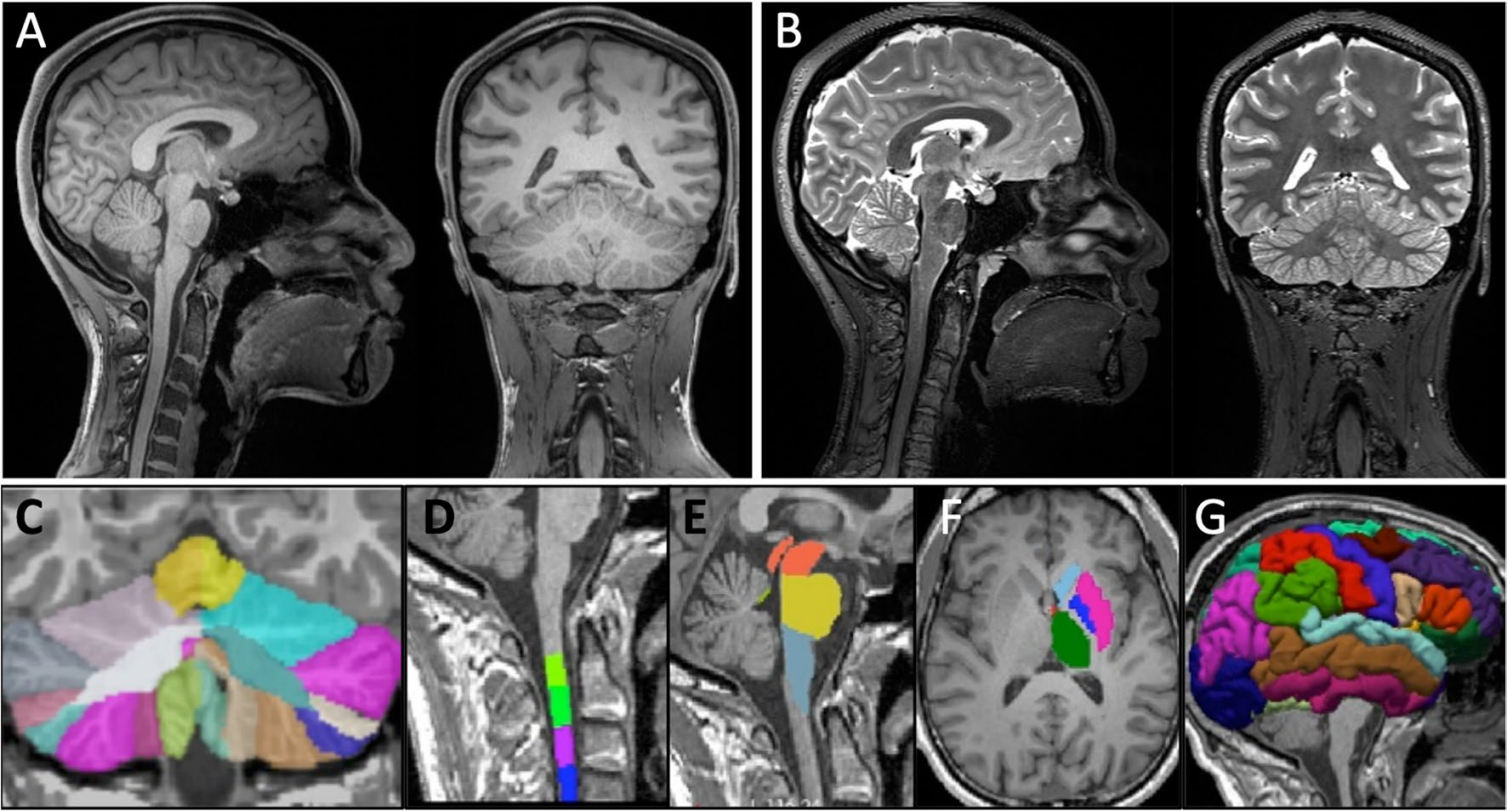
T1-weighted MPRAGE (**A**) and T2-weighted SPACE (**B**) structural MRI (0.8 mm isotropic voxels) from a healthy volunteer acquired in the sagittal orientation using the recommended protocol on a 3T scanner. Automated parcellations of T1-weighted data for quantification of volume in ataxia-relevant regions are depicted for the cerebellum (**C**; CERES Toolbox), cervical spinal cord (**D**; Spinal Cord Toolbox), brainstem (**E**; FreeSurfer), basal ganglia (**F**; FreeSurfer), and the cerebral cortex (**G**; FreeSurfer)

Our recommendations for structural MR acquisitions in patients with ataxia are given in Table 2. Briefly, we recommend acquiring high-resolution isotropic gradient-echo 3D images with 0.8–1 mm isotropic voxel size (Fig. 1), given that high-resolution imaging is even more critical for the cerebellum with its tightly folded folia and ~ 3-times thinner cortex than the cerebrum [16]. To achieve full brain coverage (FoV ~ 170 cm in the superior-to-inferior direction), at least 208 or 176 contiguous slices should be acquired for 0.8 mm or 1 mm isotropic resolution, respectively, on a sagittal acquisition plane. Although 3D images allow for multi-planar reconstructions, and therefore the evaluation of all three orthogonal planes from a single acquisition regardless of the acquisition plane, we recommend sagittal acquisition to ensure inclusion of the entire cerebellum as well as the upper cervical spinal cord in the FoV. Many hereditary ataxias involve spinocerebellar degeneration, which is reflected, for example, in the term spinocerebellar ataxias (SCAs). The inclusion of this portion of the spinal cord is of general interest, and not restricted to ataxias known to present with prominent volume loss in the spinal cord, such as Friedreich’s ataxia (FRDA) and autosomal recessive spastic ataxia of Charlevoix-Saguenay (ARSACS) [17, 18].

**Table 2:** Guidelines for AGI structural MRI protocol

Sequence	For T1w volume: MPRAGE, SPGR, or TFE, depending on vendorFor T2w volume: SPACE, CUBE, or VISTA, depending on vendor
Slice orientation	Sagittal
Voxel resolution (mm^3^)	0.8 × 0.8 × 0.8 (1 × 1 × 1 acceptable although less desirable)
Number of slices	Minimum 208 for 0.8 mm isotropic resolution (min. 176 for 1 mm isotropic resolution)
Matrix size	320 × 320 for 0.8 mm isotropic resolution (256 × 256 for 1 mm isotropic resolution)
TE/TR/TI (ms)	T1w: 2.1/2400/1000 T2w: 560/3200/- (These are starting values; adjust within an approximate range of ± 20% around these values based on scanner specifications)
FA (°)	T1w: 9 (8–10 permitted to accommodate diverse scanner hardware) T2w: variable
Phase encoding direction	Anterior-to-posterior
Acceleration	Parallel imaging (e.g., GRAPPA) in phase encoding direction (*R* = 2, 32 reference lines)

*T1w*, T1-weighted; *T2w*, T2-weighted; *MPRAGE*, Magnetization Prepared RApid Gradient Echo; *SPGR*, SPoiled Gradient-Recalled; *TFE*, Turbo Field Echo; *SPACE*, Sampling Perfection with Application-optimized Contrasts using different flip angle Evolution (Siemens); *CUBE*, not an abbreviation (GE); *VISTA*, 3D Volume ISotropic Turbo spin-echo Acquisition (Philips); *TE*, echo time; *TR*, repetition time; *TI*, inversion time; *FA*, flip angle; *GRAPPA*, GeneRalized Autocalibrating Partial Parallel Acquisition

Neuroanatomical outcomes most commonly relevant to ataxias include volumes of the cerebellar compartments including lobes and lobules (i.e., cerebellar parcellation), brainstem, basal ganglia, and, in some cases, cerebral volumes or cortical thickness and spinal cord cross-sectional area (Fig. 1). In particular, volume loss of the cerebellar grey matter and underlying white matter, generally weighted to specific sub-regions in different diseases, is widely reported in ataxias [3]. Importantly, these measures are sensitive to longitudinal changes in symptomatic and presymptomatic patients [19–21]. The anatomical and temporal profile of cerebral involvement is variable across different diseases, but a growing body of quantitative research indicates that most degenerative ataxias involve some degree of cerebral cortical and/or subcortical atrophy, as well as white matter volume loss [5, 22, 23].

Several tools for quantitative volumetric analyses using structural MRI data are available and widely used in academic research settings, including FreeSurfer (https://surfer.nmr.mgh.harvard.edu/) for cerebral cortical thickness and subcortical/brainstem parcellation, and FMRIB Software Library (FSL, https://fsl.fmrib.ox.ac.uk/fsl/fslwiki) and Statistical Parametric Mapping (SPM, https://www.fil.ion.ucl.ac.uk/spm/) for whole-brain voxel-based morphometry. In addition, specialized tools have been developed and validated for lobular segmentation of the cerebellum, including in the presence of atrophy, allowing for increasingly detailed assessments of localized morphological changes [24–27]. Notably, although the thickness of the *cerebral* cortex is now a widely reported outcome measure in MRI studies, accurate and reliable quantification of *cerebellar* cortical thickness is not yet possible using current tools and standard image resolutions due to the much thinner and more complex anatomy of the cerebellar grey matter. Automated tools for the assessment of spinal cord volumes, such as the Spinal Cord Toolbox [28], are also promising additions to the toolkit available to the ataxia imaging community.

## MR Spectroscopy

MRS allows non-invasive quantification of high-concentration (~ mM) endogenous neurochemicals [29]. These neurochemicals may be markers of aspects of the neurodegenerative pathology beyond tissue loss, such as neuronal viability, gliosis, membrane turnover, oxidative stress, and energy deficits [30]. The MRS community has recently put forth guidelines for both acquisition [31, 32] and analysis [33] of MRS data for clinical research, which we endorse. For data acquisition in ataxias (Table 3), we recommend the use of single-voxel spectroscopy with voxel-based *B*_0_ and *B*_1_ calibrations to achieve high data quality in the challenging brain regions affected, namely the cerebellum, brainstem, and spinal cord [30]. For consistent VOI prescription across subjects and scanning sessions, we recommend the use of automated tools when available [34]. Otherwise, tools such as AutoAlign (Siemens) and SmartExam (Philips) can be used to save and retrieve VOI information in longitudinal scans of the same subject. At 3 T and higher fields, the use of pulse sequences such as semi-LASER is recommended to minimize chemical shift displacement artifacts [31, 32]. Notably, a semi-LASER sequence with an optimized gradient and timing scheme has been harmonized across the major MR scanner vendors [35]. The use of short echo times is recommended to allow quantification of metabolites beyond singlet resonances (*N*-acetylaspartate, creatine, choline), such as glutamate and glutamine. Operator intervention during the acquisition should be minimized using automated methods that ensure consistency of *B*_0_ and *B*_1_ calibrations across subjects [36]. The use of optimized pulse sequences with consistent calibrations across scanning sessions allows high test–retest reproducibility of the major metabolites in spectra collected from the cerebellum over ~ 5 min, with coefficients of variance (CVs) ≤ 5% at 3 T [37]. A water reference should always be collected from the same VOI to enable concentration estimates for individual metabolites rather than ratios. Finally, saving individual transients will allow the correction of minor motion effects by frequency and phase alignment of single shots and the removal of shots that were severely affected by motion from the averaged spectrum.

**Table 3:** Guidelines for AGI MRS protocol

Single-voxel or MRSI?	Single voxel preferred due to higher achievable data quality
VOI location, size	Cerebellar WM, vermis, pons; minimum 4 mL volume
VOI selection	Use an automated VOI prescription tool if available; otherwise commercially available tools that allow collection of images in the same reference frame across subjects
Localization sequence	Semi-adiabatic LASER (sLASER), in accordance with community consensus for 3 T and higher fields [30]
TE/TR/NEX	25–30 ms/2–3 s/64–128
*B*_0_ adjustment	Adjust first‐ and second‐order shims for the targeted VOI using fully automated *B*_0_ field mapping techniques, based on 3D *B*_0_ mapping or mapping along projections
*B*_1_ adjustment	Calibrate flip angle for the targeted VOI
Water reference	Acquire unsuppressed water signal from the same VOI, with carrier frequency on water, with the same sequence as for the metabolite acquisition, but with the power for water suppression and outer volume suppression (OVS) pulses turned off (keeping the gradient scheme intact), before metabolite acquisition
Metabolite acquisition	• Evaluate water linewidth before starting metabolite acquisition, repeat *B*_0_ adjustment if linewidth is poor (> 13 Hz) • Save single shots • Evaluate water suppression efficiency, spectral linewidth and SNR during acquisition, repeat acquisition if substantial motion is detected

*VOI*, volume of interest; *WM*, white matter; *TE*, echo time; *TR*, repetition time; *NEX*, number of transients

Linear combination model fitting is recommended to estimate neurochemical concentrations, with attention to considerations outlined in detail previously [33]. The metabolites that have been most informative in ataxias include total *N*-acetylaspartate (tNAA), *myo*-inositol (mIns), and total creatine (tCr). Reductions in tNAA indicate neuronal dysfunction or loss, elevated mIns is a putative marker for gliotic activity, and elevated tCr may be a marker of gliotic activity or impairments in energy metabolism [29, 30]. In addition, a reduction in glutamate accompanied by an elevation in glutamine, observed in several ataxias [38, 39], may indicate excitatory neurotransmission deficits.

**Fig. 2:**
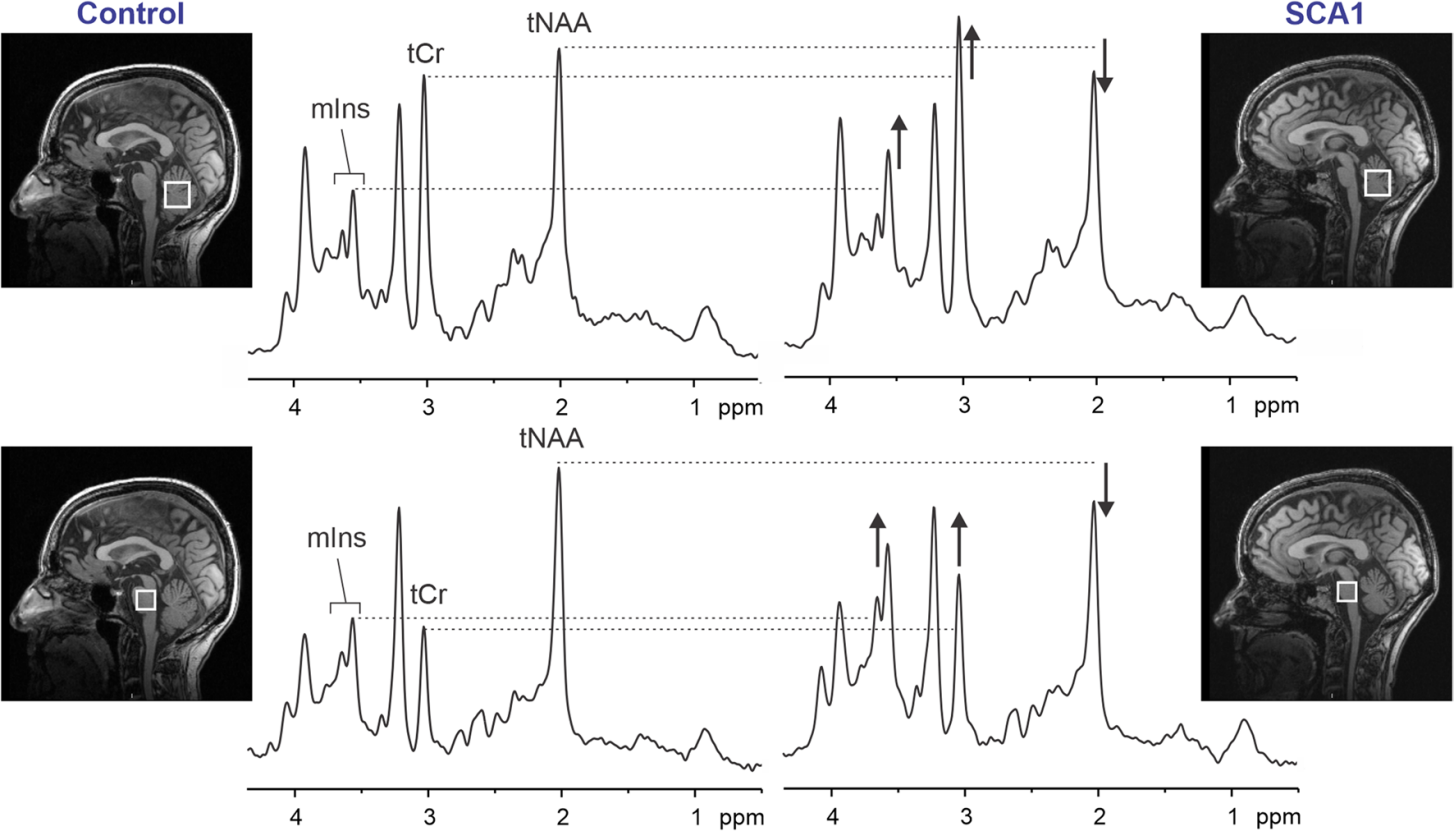
Proton MR spectra obtained from the cerebellar vermis and pons of a healthy control (left) and a patient with SCA1 (right) at 3 T (semi-LASER, TR/TE = 5000/28 ms). Voxel positions are shown in T1-weighted mid-sagittal images. Differences in the spectra from the patient vs. control in total *N*-acetylaspartate (tNAA), *myo*-inositol (mIns), and total creatine (tCr) are marked. Adapted from [85], with permission from Springer

MR spectra acquired using the recommended protocol allow the detection of neurochemical alterations in individual patients (Fig. 2), even at the preataxic stage [5, 39]. An ability to collect MRS data with reproducibly high quality when using the recommended protocol has been demonstrated in the multi-site setting [5, 40]. Importantly, the same neurochemical abnormalities were detected by different groups in SCAs [38, 41] and MRS markers were more sensitive than volumetric and diffusion metrics at the preataxic stage [5] and more sensitive to progression than a standard clinical scale [42] in SCA1.

Spinal cord MRS may also provide valuable information in ataxias with spinal cord involvement. Spinal cord MRS is generally more challenging than brain MRS due to lower signal-to-noise ratio (SNR), broader linewidth, and high sensitivity to motion. Spinal cord MRS has been reported in FRDA, with increased mIns, decreased tNAA, and a corresponding nearly twofold lower tNAA/mIns ratio in patients compared to controls [18]. The guidelines provided in Table 3 for brain MRS are broadly applicable to spinal cord MRS, with some adjustments, such as a higher number of transients (NEX = 160–256), higher linewidth threshold for acceptable data (< 20 Hz), and ideally the use of metabolite cycling [43] to allow for shot-to-shot frequency and phase correction using the water peak.

## Quantitative Susceptibility Mapping

QSM is an MRI post-processing technique that measures the magnetic susceptibility distribution within an object. QSM provides an excellent complementary view of the cerebral anatomy due to its high sensitivity toward iron content and myelination [44–46]. Data should be acquired at 3 T with a dedicated head coil with at least 32 receiver channels. QSM relies on phase images of T2*-weighted gradient-echo (GRE) acquisitions as these reflect the magnetic field distribution primarily introduced by the underlying magnetic susceptibility. The optimum phase contrast is achieved for an echo time (TE) equal to the tissue’s effective transverse relaxation time (T2*) [47]. As a variety of tissue types with different T2* values are collected by MRI in vivo, we recommend using a multi-echo GRE sequence for QSM. As a trade-off between sensitivity to susceptibility-induced field perturbations, SNR, and acquisition speed, we recommend acquiring four echoes with a rather long monopolar echo readout (bandwidth = 200–260 Hz/px). The longest TE should be between 20 and 25.5 ms, with a repetition time (TR) of 30 ms or less. The selected echo times may vary depending on the MR scanner (Table 4). We recommend the use of isotropic voxels ranging between 0.8 and 1 mm. The use of isotropic voxels minimizes the bias due to variations in the orientation of the FoV, provides the possibility to reconstruct oblique slices via multi-planar reformatting, and allows for spatial normalization into a common space (e.g., Montreal Neurological Institute (MNI) space) facilitating the application of voxel-based analysis approaches. We recommend using acquisition times of less than 8 min to minimize the vulnerability toward patient motion. Hence, we propose a transverse-oblique slab orientation with a rotation of approximately 10° to 20° around the anterior commissure–posterior commissure (AC–PC) line (readout encoding: anterior–posterior; phase encoding: right-left) to cover the whole brain efficiently (Supplementary Fig. 1). With a minimum slab thickness of 140 mm, such angulation allows whole brain coverage, including the cerebellum, across subjects with varying head sizes and anatomy with fewer number of slices and consequently shorter scan time. Because of the large susceptibility variations in the direct vicinity of the cervical spine, measuring susceptibility in the spinal cord is challenging and not yet performed regularly.

**Table 4:** Guidelines for AGI QSM protocol

Sequence	3D multi-echo gradient-echo sequence
FoV/matrix/voxel size	Transverse-oblique slab orientation. Slab thickness minimum 140 mm. FoV ca. 220 mm (read). Pixel dimensions: 0.8–1 mm isotropic. Slice oversampling: 7–11%. Rectangular FoV with FoV (phase) 75–85%, phase encoding direction: right-left
TE/TR	4 echoes with monopolar readout, the 4 echoes should be distributed evenly, TE_1_ = 3–4.5 ms,$\Delta$TE = 5.7–7 ms, 20.1 ms$\le$TE_4_$\le$25.5 ms, TR should be set as short as possible depending on the chosen repetition times, TR$\le$30 ms
Flip angle, bandwidth	13°–15°, BW_1-4_ = 200–260 Hz/px, lower bandwidths are preferred
*B*_0_ adjustment	Adjust first‐ and second‐order shims for the targeted FoV using fully automated *B*_0_ field mapping techniques [B0-shim-modus: extended (if possible, otherwise: standard)]
Acceleration	Parallel imaging in phase encoding direction (*R* = 2, 48 reference lines), partial Fourier imaging of 6/8 in slice encoding direction, elliptical sampling
Notes	Monopolar echo readout, switch on magnitude and phase images as output; for Siemens scanners, the SWI switch needs to be turned off; channel combination should be adaptive combine or SENSE (channel combination via sum of squares is not allowed); the echo times can vary depending on the gradient performance; slab orientation: starting from a transverse slab, angulate the FoV in the sagittal view to cover the whole brain including the cerebellum [typical angulation: 10°–20° relative to the AC–PC line]; flow compensation would be preferable but is not necessary

*FoV*, field of view; *TE*, echo time; *TR*, repetition time; *BW*, bandwidth; *R*, acceleration factor; *SWI*, Susceptibility-Weighted Imaging; *SENSE*, SENSitivity Encoding; *AC*–*PC*, Anterior Commissure–Posterior Commissure

**Fig. 3:**
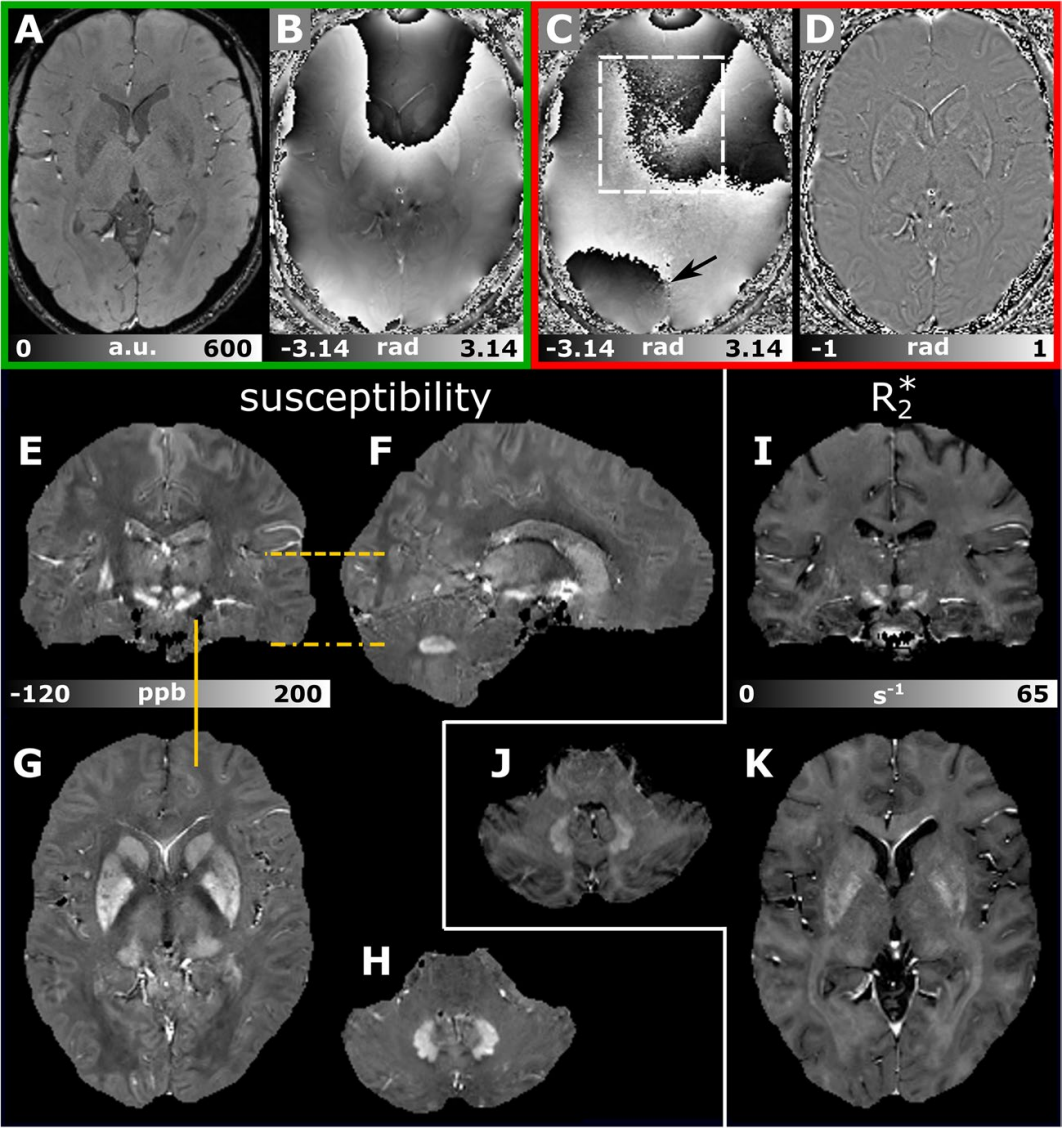
Example images of a healthy volunteer acquired with the recommended multi-echo gradient-echo imaging approach for QSM (TE_1-4_ = 3.7/9.7/15.8/21.9 ms, BW_1-4_ = 240 Hz/px, TR = 27 ms, FA = 15°, isotropic voxel size: 0.9 mm, TA = 7:16 min:s). Magnitude and raw phase images collected at echo time 21.9 ms are shown in **A** and **B**, respectively. Corresponding phase images to **B** but unsuited for QSM are shown in **C** and **D**. In **C**, the combination of multiple receiver channels yielded severe noise (dashed rectangle) and unphysically open-ended fringe lines (arrow). A high-pass filtered phase image typically obtained in susceptibility-weighted imaging is presented in **D**. Axial susceptibility (**G**, **H**) and R2* maps (**J**, **K**) at the level of the basal ganglia (**G**, **K**) and dentate nucleus (**H**, **K**) are presented. **E** and **F** show additional coronal and sagittal views of the susceptibility maps, respectively, and **I** shows the coronal R2* map. The dashed orange lines indicate the locations of the axial sections, whereas the orange line highlights the location of the sagittal section

Both the magnitude and unprocessed phase images are required (Fig. 3A, B). To compute the local magnetic field variation within an object, no frequency-varying filter (e.g., high-pass filter, as typically applied for susceptibility-weighted imaging [48] (Fig. 3D)) should be applied. In addition, special care should be granted when choosing the algorithm for the combination of the independent receiver channels. Adaptive combination on Siemens systems and SENSE-based combination on Philips systems provide artifact-free phase images, while channel combination via sum of squares produces corrupted phase image unsuited for QSM (Fig. 3C). The typical processing steps for QSM include (i) estimation of the magnetic field map, (ii) computation of the local magnetic field by removing magnetic field contributions originating from magnetic sources outside of the object (i.e., the brain), and (iii) solving the inverse-problem to convert the local magnetic field to the underlying magnetic susceptibility. More details on QSM processing can be found in recent reviews [45, 49]. Generating QSM maps currently relies on offline data processing (i.e., not on the scanner), with multiple software packages available to the research community (https://www.emtphub.org/magnetic-software-packages/).

The recommended multi-echo GRE imaging protocol also allows the calculation of the effective transverse relaxation rate (R2*), a quantitative measure that sensitively indicates the degree of magnetic field inhomogeneity at a microscopic scale, by analyzing the magnitude signal decay [50]. Algorithms for R2* mapping are typically available directly on MR scanners or as part of offline software packages (see above). Similar to magnetic susceptibility, R2* also correlates linearly with iron in deep grey matter [51, 52], while in white matter, myelin and iron substantially contribute to both magnetic susceptibility and R2*. However, these two measures can provide complementary information leading to a more detailed assessment of tissue composition [44, 53, 54] (Fig. 3E–K).

QSM has been utilized to study the iron concentration in deep grey matter in different ataxias using in-plane resolutions ≤ 1 mm. Higher susceptibilities were measured in the substantia nigra and dentate nucleus in small groups of patients with the cerebellar type of multiple system atrophy (MSA-C) compared to matched controls, indicating higher iron concentration [55, 56]. Higher magnetic susceptibilities indicating increased iron concentration were also found in the globus pallidus, red nucleus, and substantia nigra in patients with SCA3 [57]. The excellent depiction of deep grey matter on susceptibility maps also allows for quantification of atrophy of these structures. For instance, atrophy of dentate nuclei has been demonstrated in patients with different ataxias, including SCA6 and FRDA [56, 58, 59]. Consequently, integrating a high spatial isotropic resolution (≤ 1 mm) multi-echo GRE scan into the MRI protocol allows for voxel-based statistics of volumes, susceptibilities, and R2*, opening the door to identifying disease-related patterns.

## Diffusion MRI

dMRI [60] relies on the anisotropic diffusion of water molecules in organized tissues, such as the brain white matter or spinal cord, to recover microstructural and connectivity information through local biophysical models and tractography [61]. Axonal membranes and myelin hinder the diffusion process [62] and constitute the primary source of white matter signal in dMRI, thereby providing contrasts sensitive to neurodegeneration. This phenomenon can be quantified by taking measurements along multiple orientations, called *diffusion gradients*, and diffusion weightings, summarized in the so-called *b*-*value* [63]. Among biophysical models [64, 65] used to characterize the dMRI signal at each voxel, diffusion tensor imaging (DTI) [66] is the most widely used technique.

Recommendations for dMRI in ataxias are given in Table 5 with flexibility in acquisition parameters to accommodate the widely varying capabilities of MR scanners. Data acquisition should be performed at 3 T using a multi-channel receive array with at least 32 channels. Two-dimensional spin-echo echo-planar imaging (SE-EPI) should be used to cover the whole brain, including the cerebellum, with axial slices. We recommend isotropic voxels in the range of 1.5 to 2 mm with minimum superior-inferior coverage of about 140 mm (i.e., 70 to ~ 92 slices). If collected together with QSM and rs-fMRI in the same session, the same FoV should be used for all acquisitions, which will typically require 10–20° angulation relative to the AC–PC line for whole brain coverage, including the entire cerebellum, consistently across subjects when using a 140 mm slab (Supplementary Fig. 1). If angulation of the dMRI slab is not feasible on the scanner, the number of slices should be increased to ensure whole cerebellum coverage.

**Table 5:** Guidelines for AGI diffusion MRI protocol

Sequence	Spin-echo echo-planar imaging (SE-EPI), diffusion-weighted
Geometry	1.5–2 mm isotropic voxels. Axial slices, minimum superior-inferior coverage = 140 mm (e.g., 70 slices × 2 mm), FoV typically tilted 10°–20° relative to the AC–PC line to ensure whole brain coverage, including the entire cerebellum. Interleaved, contiguous slices (no gap)
TR/TE/NEX	TR = minimum available, typically 3000–10,000 ms TE = minimum available, typically 60–90 ms NEX = 32 volumes/directions or more (see *q-space* section)
Acceleration	Multi-slice acceleration = 3 to 4 and/or phase encoding acceleration (e.g., GRAPPA) = 2 to 3 Multi-slice is preferred if available
*q-space*	At least 32 directions with *b*-value = 1000–1500 s/mm^2^, plus 3 volumes at *b* = 0 s/mm^2^ with phase encoding: anterior-to-posterior and 3 volumes at *b* = 0 s/mm^2^ with phase encoding: posterior-to-anterior Alternatively, and if possible, repeat the 32 (or more) directions with 3 volumes at *b* = 0/mm^2^ with phase encoding: posterior-to-anterior Additional *b*-shells (e.g., *b* = 500 s/mm^2^, 2000s/mm^2^, 3000 s/mm^2^) are recommended when multi-slice acceleration is available

*FoV*, field of view; *AC–PC*, anterior commissure–posterior commissure; *TR*, repetition time; *TE*, echo time; *NEX*, number of acquisitions; *GRAPPA*, GeneRalized Autocalibrating Partial Parallel Acquisition

Imaging acceleration is strongly recommended if available, using multi-slice EPI up to fourfold and/or parallel imaging (e.g., GeneRalized Autocalibrating Partial Parallel Acquisition (GRAPPA), SENSitivity Encoding (SENSE)) up to threefold [67]. However, care should be taken when selecting factors for multi-slice and in-plane acceleration as over-accelerating may negatively affect image quality (lower SNR, artifacts). It is also particularly important to ensure that multi-channel reconstruction is done using SENSE [68].

We recommend acquiring at least 32 diffusion gradient directions with a *b*-value of 1000–1500 s/mm^2^ and 3–4 additional volumes with *b* = 0 s/mm^2^. Multi-shell acquisitions that include a larger number of gradient directions sampled across multiple *b*-values (e.g., 500 s/mm^2^ and 2000s/mm^2^) are recommended when multi-slice acceleration is available. Diffusion gradient vectors should be defined using an incremental table [69], which can be generated using online tools (https://github.com/mandorra/multishell-qspace-gradients, https://www.massive-data.org/massive-data#h.cytj3ar4i2v) or replicated based on existing protocols [11, 67]. At a minimum, we recommend obtaining the *b* = 0 s/mm^2^ data twice with opposite phase encoding directions (usually A > > P and P > > A) to correct for geometric distortions; where feasible, the full dataset can be acquired in each encoding direction to improve SNR [70].

**Fig. 4:**
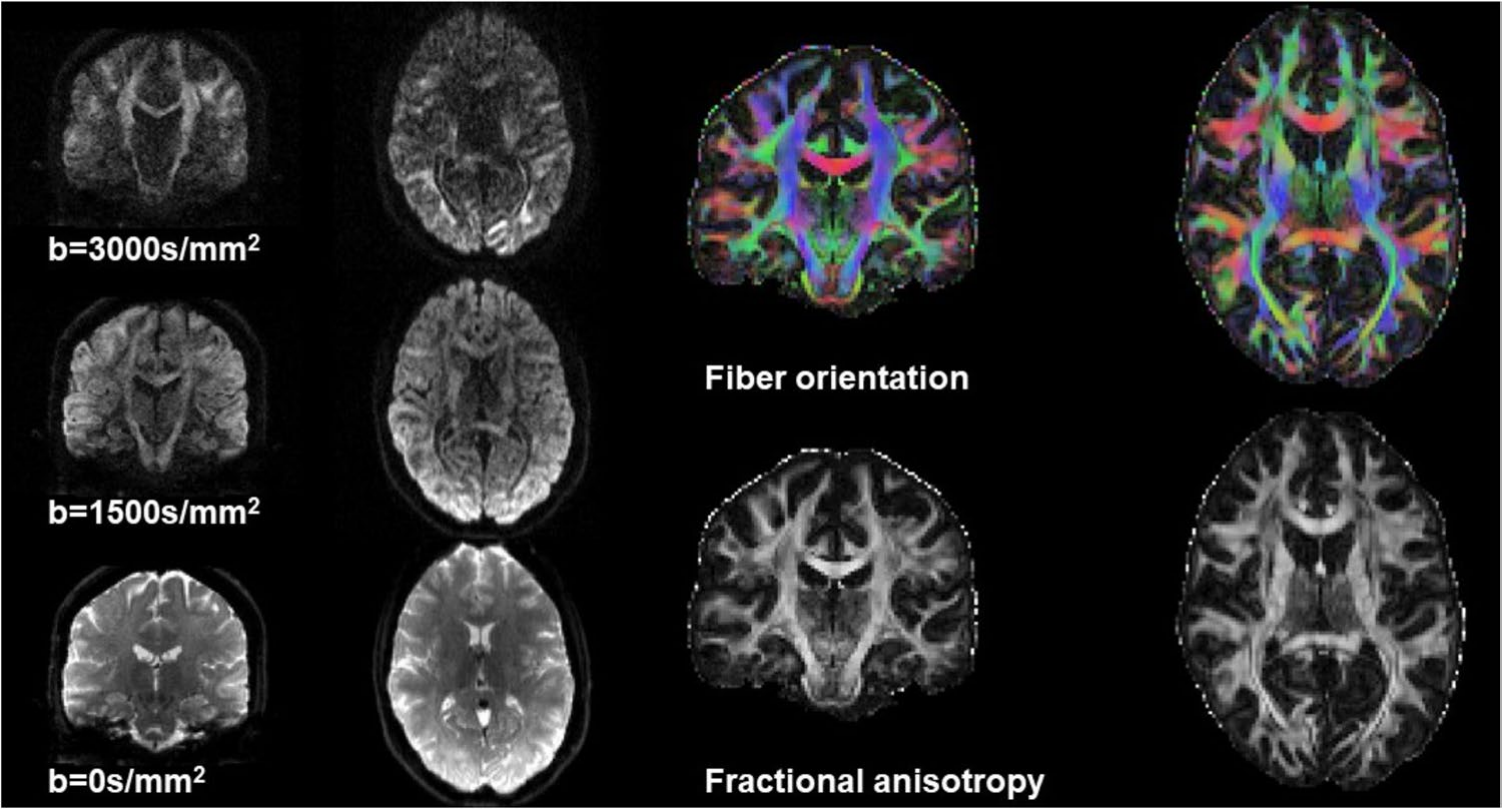
Example diffusion MRI data obtained with the recommended MRI protocol. The left panel shows examples of *b* = 0 s/mm^2^, *b* = 1500 s/mm^2^, and *b* = 3000 s/mm^2^ images after preprocessing. The right panel shows the corresponding fractional anisotropy map and primary fiber orientation from the diffusion tensor

Prior to extracting quantitative metrics from dMRI data, distortions primarily caused by magnetic field inhomogeneities need to be corrected. These include susceptibility-induced distortions that arise from head geometry and are largely constant for a given subject, and eddy current-induced distortions that result from rapidly switching diffusion gradients and are unique to each diffusion-weighted image. Additionally, head motion between and within images needs to be corrected. While between-image motion is more common and easily accounted for by rigid transformations, within-image motion results in low intensity and misaligned slices. Software packages are available (e.g., FSL, https://fsl.fmrib.ox.ac.uk/fsl/fslwiki/; MRtrix, https://www.mrtrix.org/; TORTOISE, https://tortoise.nibib.nih.gov/) to perform these preprocessing steps, as well as image denoising, removal of Gibb’s ringing artifacts, estimation of diffusion metrics, and tractography. The most widely used metrics to assess possible degeneration of axonal pathways are obtained from the DTI model and include fractional anisotropy (FA), axial, radial and mean diffusivities, and primary fiber orientation (Fig. 4). A decrease in FA is interpreted as altered white matter microstructure (e.g., axonal loss, demyelination) and is typically accompanied by an increase in diffusivity. Other more advanced diffusion metrics such as fiber density and cross-section from fixel-based analysis [71] can be used to better characterize individual fiber bundles, even in regions with complex (i.e., crossing) white matter configurations. Similarly, recent biophysical multi-compartment models [64] can be used to extract metrics that are more specific to microstructural characteristics such as axonal diameter, density, and dispersion.

The AGI dMRI protocol is flexible enough so that many of the above-mentioned diffusion metrics can be obtained. Widespread white matter damage has been demonstrated in SCAs using the proposed dMRI protocol, including at the preataxic stage [5, 72, 73]. Furthermore, dMRI metrics have been shown to detect the progression of microstructural changes with high sensitivity [21]. Finally, two large international consortia, READISCA [5] for SCA1 and SCA3 and TRACK-FA [6] for FRDA, are currently using the proposed AGI dMRI protocol for clinical trial readiness studies.

## Resting-State Functional MRI

Functional magnetic resonance imaging (fMRI) is sensitive to subtle changes in local blood oxygenation that result from neurovascular coupling. Changes in the fMRI signal can be experimentally induced (i.e., “task-based” fMRI), or measured as the unconstrained, spontaneous fluctuations of the blood-oxygen-level-dependent (BOLD) signal over time (i.e., “resting-state” fMRI, rs-fMRI) [74]. The AGI protocol focusses only on rs-fMRI, due to its broad generalizability across scanners and experimental contexts. However, the same acquisition parameters are generally appropriate for both contexts. The recommended fMRI protocol has wide latitude in acquisition parameters to accommodate the widely varying capabilities of MR scanners (Table 6).

**Table 6:** Guidelines for AGI (resting-state) functional MRI protocol

Sequence	Gradient-echo echo-planar imaging (GRE-EPI), BOLD-weighted
Geometry	2–3 mm isotropic voxels. Axial slices, minimum superior-inferior coverage = 140 mm (e.g., 70 slices × 2 mm), FoV typically tilted 10°–20° relative to the AC–PC line to ensure whole brain coverage, including the entire cerebellum. Interleaved, contiguous slices (no gap)
TR/TE/NEX	TR = use minimum available, typically between 500 and 3000 ms (see acceleration, below) TE = 30 to 40 ms NEX = 200–1000 volumes (at least 10 min of continuous acquisition)
Acceleration	Multi-slice acceleration = 4 to 8 and/or phase encoding acceleration (e.g., GRAPPA) = 2 to 3 Multi-slice is preferred if available
Notes	Phase encoding direction: anterior-to-posterior Additional short (30 s) acquisition using reverse phase encoding (posterior-to-anterior) **OR** a gradient field map must be acquired for image distortion correction Eyes open with fixation on a cross in the center of the visual field (no video or audio to the participant)

*BOLD*, blood-oxygen-level-dependent; *FoV*, field of view; *AC–PC*, anterior commissure–posterior commissure; *TR*, repetition time; *TE*, echo time; *NEX*, number of acquisitions; *GRAPPA*, GeneRalized Autocalibrating Partial Parallel Acquisition

The fMRI acquisition should consist of 2D gradient-recalled echo–echo-planar imaging (GRE-EPI) volumes acquired in the axial plane. Voxel sizes should be in the range of 2–3 mm isotropic with at least 140 mm of superior-inferior coverage (e.g., 2 mm × 70 slices) and the same FoV angulation relative to the AC–PC line as QSM and dMRI ensuring whole cerebellum coverage (Supplementary Fig. 1). Slices should be contiguous and interleaved to minimize excitation cross-talk between neighboring slices [75]. No less than 10 min of data should be acquired to ensure the reliability of connectivity quantification [76, 77]. An additional 30-s acquisition with opposite phase encoding (e.g., main acquisition A > > P, additional acquisition P > > A) *or* a gradient-recalled echo field map with the same geometry should be acquired for image distortion correction. Multi-slice (up to × 8) or phase acceleration (e.g., GRAPPA × 2–3) is recommended where available to increase temporal resolution. Participants should be instructed to keep their eyes open and look at a fixation cross that is presented roughly in the center of their visual field to maximize quantification reliability [78], and minimize the likelihood of participants falling asleep, which confounds the signal [79]. No other visual or auditory stimuli (videos, music, etc.) should be provided. rs-fMRI is most commonly used to investigate brain functional connectivity [80], although to date it has been researched less in ataxias compared to other neurological diseases. Functional connectivity is quantified as the strength of the correlation in the fMRI time series recorded in discrete brain regions. Stronger correlations reflect greater information sharing or synaptic coupling between regions. Importantly, this need not be directly reflective of underlying structural pathways, as functional connectivity between two regions may be mediated through multi-synaptic pathways. There is a large range of rs-fMRI analysis approaches available that generate summary outcome measures that can be statistically compared between a patient group and a control group to assess brain network integrity. Several software packages such as Analysis of Functional Neuro Images (AFNI; http://afni.nimh.nih.gov/afni), the CONN toolbox (https://www.nitrc.org/projects/conn/), MELODIC (https://fsl.fmrib.ox. ac.uk/fsl/fslwiki/MELODIC), and Group ICA of fMRI Toolbox Software (GIFT; http://mialab.mrn.org/software/gift/) are commonly used to process and analyze rs-fMRI data.

Seed-to-seed or seed-to-voxel approaches respectively investigate connectivity between a small number of predefined regions, or between a predefined region and the whole brain. Appropriate statistical correction to account for multiple comparisons must be undertaken in these cases. As an example of this approach, Cocozza and colleagues [81] used a seed-to-seed approach to demonstrate reduced cerebro-cerebellar and increased cerebro-cerebral connectivity in participants with FRDA relative to healthy controls. A larger number of seeds can also be defined using atlases that segment the brain into anywhere from tens to hundreds of regions. In this case, a 2 × 2 matrix (also known as a graph) can be generated by calculating the connectivity between all possible region pairs. The mathematical properties (i.e., graph metrics) of the network can then be calculated. Chen and colleagues [82] used a graph analysis approach to show that functional network structure is reorganized in people with SCA3 relative to healthy controls. Another example of this approach is the work of Jiang et al. [83], who used a graph metric, in combination with another complimentary analysis approach, to quantify functional disruptions in atrophied regions in people with sporadic adult-onset ataxia. Finally, independent components analysis (ICA) is another common way to investigate functional connectivity. ICA detects sets of brain regions that have a similar time course of activity across the fMRI acquisition period, identifying whole-brain intrinsic functional networks. Van der Horn and colleagues [84] recently employed an ICA analysis to identify a network of brain regions encompassing the cerebellum, anterior striatum, and fronto-parietal cortices that are implicated in SCA3. rs-fMRI is not used clinically in ataxia contexts, and its utility as a prospective imaging biomarker or outcome measure in clinical care or trial contexts remains to be validated. However, a growing body of evidence in ataxias, and extensive analogous work in other progressive neurodegenerative diseases, supports its utility in characterizing whole-brain, systems-level dysfunction in degenerative ataxias.

## Conclusions

Early and accurate evaluation of brain and spinal cord atrophy, neurochemistry, microstructure, susceptibility, and resting-state function in degenerative ataxias represents important targets for future therapeutic interventions that aim to halt neurodegeneration and promote neuroprotection. A prescriptive but flexible MRI protocol that can be widely adopted across clinical and research sites globally will facilitate increased opportunities for prospective and retrospective multi-site data aggregation and provide a common platform for validating and implementing quantitative MRI measures into clinical care and trial settings.

## Members of the AGI MR Biomarkers Study Group

Astrid Adarmes-Gómez^1, 2^, Andreas Thieme^3^, Kathrin Reetz^4, 5^, Marcin Rylski^6^, Thiago JR Rezende^7^, Vincenzo A. Gennarino^8, 9, 10, 11, 12^, Eva-Maria Ratai^13^, Caterina Mariotti^14^, Anna Nigri^15^, Lorenzo Nanetti^14^, Martina Minnerop^16, 17, 18^, Sylvia Boesch^19^, Elisabetta Indelicato^19^, Chiara Pinardi^15, 20^, Kirsi M Kinnunen^21^, Niccolo Fuin^21^, Alexander Gussew^22^, Cherie Marvel^23^, James Joers^24^.

## Study Group Members Affiliations – AGI MR Biomarkers Study Group 

1 Instituto de Biomedicina de Sevilla, Hospital Universitario Virgen del Rocío/CSIC/Universidad de Sevilla, Sevilla, Spain.

2 Centro de Investigación Biomédica en Red sobre Enfermedades Neurodegenerativas (CIBERNED), Madrid, Spain.

3 Department of Neurology and Center for Translational Neuro- and Behavioral Sciences (C-TNBS), Essen University Hospital, University of Duisburg-Essen, Essen, Germany.

4 Department of Neurology, RWTH Aachen University, Aachen, Germany.

5 JARA Institute Molecular Neuroscience and Neuroimaging, Forschungszentrum Jülich GmbH and RWTH Aachen University, Aachen, Germany.

6 Department of Radiology, Institute of Psychiatry and Neurology (IPiN), Warsaw, Poland.

7 Department of Neurology, School of Medical Sciences, University of Campinas (UNICAMP), Campinas, Brazil.

8 Department of Genetics & Development, Columbia University Irving Medical Center, New York, NY, USA.

9 Columbia Stem Cell Initiative, Columbia University Irving Medical Center, New York, NY, USA.

10 Department of Pediatrics, College of Physicians & Surgeons, Columbia University Irving Medical Center, New York, NY, USA.

11 Department of Neurology, Columbia University Irving Medical Center, New York, NY, USA.

12 Initiative for Columbia Ataxia and Tremor, Columbia University Irving Medical Center, New York, NY, USA.

13 Massachusetts General Hospital, Department of Radiology, Harvard Medical School, A. A. Martinos Center for Biomedical Imaging, Charlestown, MA, USA.

14 Fondazione IRCCS Istituto Neurologico Carlo Besta, Milan, Italy.

15 Neuroradiology Unit, Fondazione IRCCS Istituto Neurologico Carlo Besta, Milan, Italy.

16 Institute of Neuroscience and Medicine (INM-1), Research Centre Juelich, Juelich, Germany.

17 Institute of Clinical Neuroscience and Medical Psychology, Medical Faculty & University Hospital Düsseldorf, Heinrich Heine University Düsseldorf, Düsseldorf, Germany.

18 Department of Neurology, Center for Movement Disorders and Neuromodulation, Medical Faculty & University Hospital Düsseldorf, Heinrich Heine University Düsseldorf, Düsseldorf, Germany.

19 Center for Rare Movement Disorders Innsbruck, Department of Neurology, Medical University of Innsbruck, Innsbruck, Austria.

20 Health Physics Unit, ASST Nord Milano, Milan, Italy.

21 IXICO, London, UK.

22 University Clinic and Outpatient Clinic for Radiology, University Hospital Halle (Saale), Halle (Saale), Germany.

23 Department of Neurology, Johns Hopkins University School of Medicine, Baltimore, MD, USA.

24 Center for Magnetic Resonance Research, Department of Radiology, University of Minnesota, Minneapolis, MN, USA.

### Electronic supplementary material

Below is the link to the electronic supplementary material.

Supplementary file1 (DOCX 435 KB)

## Data Availability

Example protocols that comply with the recommendations of the AGI MR Biomarkers working group can be found at the Open Science Framework: https://osf.io/af46y/?view_only=82d605af57ec477b9ca8ba8f2404239c
