# Correction to: MR Imaging in Ataxias: Consensus Recommendations by the Ataxia Global Initiative Working Group on MRI Biomarkers

**DOI:** 10.1007/s12311-023-01589-3

**Published:** 2023-08-15

**Authors:** Gülin Öz, Sirio Cocozza, Pierre-Gilles Henry, Christophe Lenglet, Andreas Deistung, Jennifer Faber, Adam J. Schwarz, Dagmar Timmann, Koene R. A. Van Dijk, Ian H. Harding, on behalf of the AGI Working Group on MRI Biomarkers, Astrid Adarmes-Gómez, Andreas Thieme, Kathrin Reetz, Marcin Rylski, Thiago JR Rezende, Vincenzo A. Gennarino, Eva-Maria Ratai, Caterina Mariotti, Anna Nigri, Lorenzo Nanetti, Martina Minnerop, Sylvia Boesch, Elisabetta Indelicato, Chiara Pinardi, Kirsi M Kinnunen, Niccolo Fuin, Alexander Gussew, Cherie Marvel, James Joers

**Affiliations:** 1 Center for Magnetic Resonance Research, Department of Radiology, University of Minnesota, 2021 Sixth Street Southeast, Minneapolis, MN 55455 USA; 2 UNINA Department of Advanced Biomedical Sciences, University of Naples Federico II, Naples, Italy; 3 Department for Radiation Medicine, University Clinic and Outpatient Clinic for Radiology, University Hospital Halle (Saale), Halle (Saale), Germany; 4 German Center for Neurodegenerative Diseases (DZNE), Bonn, Germany; 5 Department of Neurology, University Hospital Bonn, Bonn, Germany; 6 Takeda Pharmaceuticals Ltd., Cambridge, MA USA; 7 Department of Neurology and Center for Translational Neuro- and Behavioral Sciences (C-TNBS), Essen University Hospital, University of Duisburg-Essen, Essen, Germany; 8 Digital Sciences and Translational Imaging, Early Clinical Development, Pfizer, Inc., Cambridge, MA USA; 9 Department of Neuroscience, Central Clinical School, Monash University, Melbourne, Australia; 10 Monash Biomedical Imaging, Monash University, Melbourne, Australia

**Correction to: The Cerebellum**

10.1007/s12311-023-01572-y

The original version of this article unfortunately missed properly capturing the list of collaborating authors in PubMed.

The original article and PubMed listing have been corrected.

