# Supplementary material for: MR Imaging in Ataxias: Consensus Recommendations by the Ataxia Global Initiative Working Group on MRI Biomarkers
Source: Cerebellum. 2023 Jun 6;23(3):931–45. doi: 10.1007/s12311-023-01572-y (PMC11102392; doi:10.1007/s12311-023-01572-y)

**SUPPLEMENTARY MATERIAL:**

**MR Imaging in Ataxias: Consensus Recommendations by the Ataxia Global Initiative Working Group on MRI Biomarkers**

**Supplementary Fig. 1:** Example field-of-view (FoV) placement with a rotation of 20° around the anterior commissure - posterior commissure (AC-PC) line for advanced sequences (QSM, dMRI, rs-fMRI)
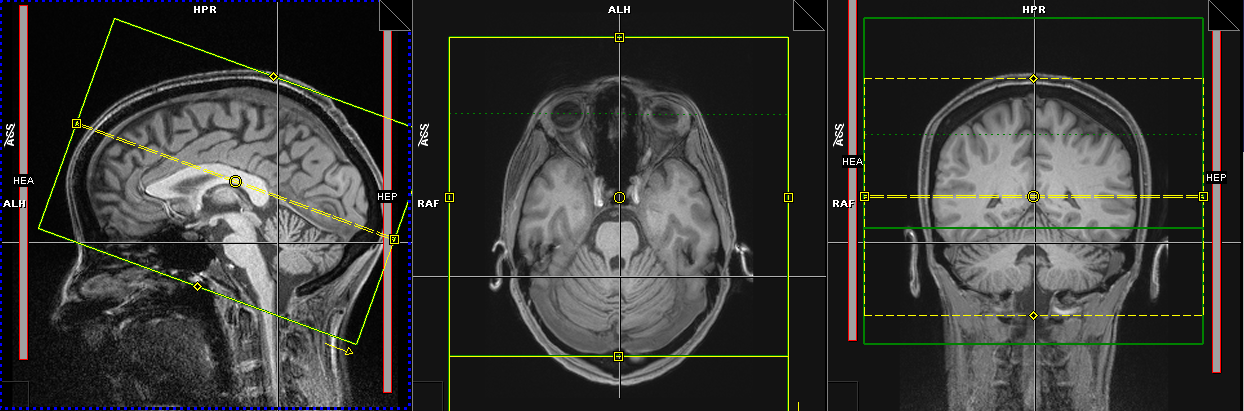

Supplement: Supplementary file 1 — Supplementary file1 (DOCX 435 KB) [file 12311_2023_1572_MOESM1_ESM.docx]
